# *Arrabidaea chica* chloroform extract modulates estrogen and androgen receptors on luminal breast cancer cells

**DOI:** 10.1186/s12906-022-03506-3

**Published:** 2022-01-20

**Authors:** Douglas C. Brandão, Paula M. A. P. Lima, Isabella C. Martins, Carina S. Cordeiro, Antonielle O. Cordeiro, Lara Vecchi, Joyce F. C. Guerra, Priscila C. Orsolin, Matheus C. Gazolla, Danilo S. Costa, Ademar A. da Silva Filho, Thaise G. Araújo

**Affiliations:** 1 Laboratory of Genetics and Biotechnology, Institute of Biotechnology, Federal University of Uberlandia, Rua Major Jerônimo, 566, Sala 601, Patos de Minas, MG 38700-002 Brazil; 2 Laboratory of Cytogenetic and Mutagenesis, University Center of Patos de Minas, Patos de Minas, MG Brazil; 3 Laboratory of Nanobiotechnology, Institute of Biotechnology, Federal University of Uberlandia, Uberlandia, MG Brazil; 4 Institute of Biotechnology, Federal University of Uberlandia, Patos de Minas, MG Brazil; 5 Faculty of Pharmacy, Department of Pharmaceutical Sciences, Federal University of Juiz de Fora, Juiz de Fora, MG Brazil

**Keywords:** Natural Products, Breast Cancer, Hormone Receptors, Hormonotherapy, Carcinogenicity

## Abstract

**Background:**

Breast Cancer (BC) is the most common cancer in women worldwide and, although 70% of patients are responsive to selective Estrogen Receptor (ER) modulators such as Tamoxifen (Tam), patients’ survival is comprised by resistance to endocrine therapy. Brazilian flora, especially the Amazon biome, is one of the richest global sources of native species with potentially bioactive compounds. *Arrabidaea chica* is a plant native to the Amazon that has been used in the treatment of different diseases. However, its action on BC remains unclear.

**Methods:**

Herein the biological effects of the chloroform extract of *A. chica* (CEAC) were evaluated on BC cells and in *in vivo* model. After confirmation of CEAC antioxidant capacity, cells were treated with CEAC and Tam, alone and with CEAC+Tam. The cell viability was evaluated by MTT and hormone receptor transcripts levels were assessed (*ESR1*, *ESR2* and *AR*). Finally, anticarcinogenicity of CEAC was recorded in *Drosophila melanogaster* through Epithelial Tumor Test (ETT).

**Results:**

The study confirmed the antioxidant activity of CEAC. CEAC was selective for MCF-7, downregulating *ESR2* and *AR* transcripts and upregulating *ESR2* expression. The modulatory effects of CEAC on ERs did not differ between cells treated with Tam and with CEAC+Tam. Interestingly, previous treatment with CEAC, followed by treatment with Tam promoted a significant decrease in cell viability. The extract also presented anticarcinogenic effect in *in vivo* assay.

**Conclusion:**

The bioassays on breast tumor cells demonstrated the antiproliferative activity of the extract, which modulated the expression of hormone receptors and sensitized luminal tumor cells to Tam. These results suggest that CEAC could be a complementary treatment for BC.

## Background

Epidemiological data on cancer are still alarming with an annual global incidence of over 439.2 new cases per 100,000 men and women per year. In 2020, there were estimated 9.9 million deaths [1, 2]. Breast cancer (BC) remains the most common non cutaneous tumor among women worldwide [3–5]. Its occurrence is steadily increasing in developing countries in which between 1 in 8 (about 12%) women will develop invasive BC during her lifetime [6, 7]. Worldwide, 2,088,849 million new BC cases and 626,679 deaths were recorded in 2018, expecting 3 million cases in 2030 [8].

BC is a hormone-related tumor [9, 10]. Therefore, nuclear receptor (NR) family of transcription factors plays essential roles in development and maintenance of malignant breast cells [11]. Estrogen receptor (ER) and Androgen receptor (AR) are frequently co-expressed in BC, although they may behave in different ways in view of the tumor heterogeneity [12]. ER-α and ER-β have similar binding affinities for estrogens; share a high degree of homology in the DNA-binding regions but interact with different other proteins suggesting divergence in transcriptional machinery control [13]. Although distinct genes codify for ER-α and ER-β, they can exist as heterodimer, and ER-β may modulate ER-α activity [14]. AR is structurally similar to ERs and how it drives, promotes or controls breast tumorigenesis remains largely unexplored [15, 16].

In this context, assessment of a breast cancer’s receptor status is essential to classify and define therapeutic strategies [17]. Accordingly, BC is categorized in four main molecular subtypes based on ER-α, progesterone receptor (PgR), and human epidermal growth factor receptor 2 (Her2) expression [11]. The tumors are divided into Luminal type expressing ER and/or PgR, being Luminal A when lack Her2, and Luminal B when express high levels of Ki67. Luminal B may or may not express Her2. Her2 enriched are BC which are negative for hormone receptors and present high levels of Her2. Finally, triple-negative BC do not express any of above markers [18, 19].

Nearly 70% of breast tumors express hormone receptors (ER and/or PgR) with better outcomes [20, 21]. Moreover, a high prevalence of ER-positive BCs express the androgen receptor (AR), and AR expression may be also associated with better outcome [22]. ER-α-positive BCs initially respond to antagonists or antiestrogens and pre- and postmenopausal women have benefited from these therapeutic strategies [23]. These tumors are mainly treated with Tamoxifen (Tam), which has been clinically used for the last 40 years [24]. Tam is a triphenylethylene derivative that functions as selective ER modulator (SERM) [25, 26]. In the breast, Tam acts as an antagonist that binds to ER and impairs estrogenic effects [27, 28]. Tam can reduce the chance of recurrence in 40 to 50% after 5 years and 30% after 10 years of treatment [29]. However, about 30% of women develop de novo or acquire resistance to hormonal therapies progressing to a metastatic disease [30, 31].

Tam resistance has been associated with the expression levels of ER-α and ER-β [32, 33] especially to lower levels of ER-β [34]. The resistance toward Tam treatment has been also related with the expression of the AR [35]. However, ER-α-negative BCs have been have been benefitted from Tam in terms of recurrence, once Tam can bind directly to AR [36]. Park and collaborators demonstrated that that ER-α-positive and AR-positive BCs display a better prognosis compared to ER-α-negative and AR-positive BCs [37].

Natural product-derived compounds are being extensively explored as potential cancer treatments, specially to overcome resistance and side effects, and to prolong patient’s overall survival [38, 39]. Considering ER-positive BC, is desirable to develop new therapeutic agents that modulate ER expression achieving greater effectiveness, with less side effects impairing thrombosis and pulmonary embolism detected in 10 years-treated patients [40, 41].

Brazilian flora, especially the Amazon biome, is one of the richest global sources of native species with potentially bioactive compounds for the treatment of various diseases. The species *Arrabidaea chica* (Humb. & Bonpl.) B. Verlot, popularly known as “pariri” “crajiru” or “chica”, has been explored and its components (flavonoids, anthocyanins, tannins and phytosterols) isolated for the fight against fungi, bacteria, inflammatory processes, and tumors. The ability of *A. chica* extracts in reducing Ehrlich tumors without adverse effects has been previously describe [42]. However, the in vitro antitumor activity of *A. chica* extracts, especially in ER-positive BC, has not been described yet. Furthermore, its potential to induce cancer has not been evaluated.

The Epithelial Tumor test (ETT) has been used to detect epithelial tumor clones (Warts) in *Drosophila melanogaster*. This test evaluates the carcinogenic, anticarcinogenic, chemopreventive, and modulatory potential of different substances [38, 43, 44]. The test stands outs for its sensibility and reliability as a toxicological test and detects the loss of heterozygosity of the *wts* tumor suppressor gene, which leads to uncontrolled cell proliferation and the consequent formation of epithelial tumors [45]. The *wts* homologous gene in humans is the Large Tumor Suppressor Kinase 1 gene (*LATS1*) [46], and *D. melanogaster* shows considerable genetic homology with humans when compared to other mammalian models [47, 48] being used in important researchers related to treatment of tumors [49].

Here, we hypothesize that *A. chica* chloroform extract (CEAC) have selective effect across BC subtypes, modulating hormone receptors and cellular response to Tam treatment. To test this notion, we evaluated the cytotoxic potential of CEAC in four BC cell lines. In ER-positive strains, we compared how the treatment with CEAC alone or with Tam altered the viability, and hormone expression in MCF-7 and T-47D cells. Finally, we evaluated the antitumor potential of CEAC in *in vivo* model.

## Methods

### Chemicals

High purity chemicals and reagents were purchased from commercial sources, and all dilutions were prepared immediately before use. For *in vitro* assays, Dulbecco’s Modified Eagle Medium Nutrient Mixture F-12 (DMEM/F-12), Roswell Parki Memorial Institute (RPMI-1640), Leibovitz’s (L-15), fetal bovine serum (FBS), Epidermal Growth Factor (EGF), insulin and antibiotic gentamicin solution were purchased from (Gibco®). The 3-[4,5-dimethylthiazole-2—yl]2,5-diphenyltetrazolium bromide (MTT), CAS 57360-69-7, dimethylsufoxide (DMSO), CAS 67-68-5 and hydrocortisone were purchased from Sigma-Aldrich®. Tamoxifen Citrate (Sandoz) was gently donated by the Cancer Hospital from Federal University of Uberlandia.

For *in vivo* assays, Doxorubicin hydrochloride (DOX), commercial name Fauldoxo® (CAS 25316-40-9, batch 19B1091, Laboratório Industrial Brasileiro de Biologia e Síntese - Libbs, São Paulo – Brazil) was used as positive control at 0.4 mM. This concentration was based on previous studies that demonstrated the induction of epithelial tumors in D. melanogaster by DOX [44, 50–53]. Tween 80 (CAS 9005-65-6) at 1% (v/v) was used as negative control and for dilution of the compounds.

### Plant material and preparation of *A. chica* extracts

Attending the Brazilian legislation (Law number 13.123 / 2015) this study was registered in the National System of Management of Genetic Heritage and Associated Traditional Knowledge under number A5573F8.

Leaves of *A. chica* were collected at the Faculty of Pharmacy’s Medicinal Herb Garden (UFJF), Juiz de Fora city, MG. The plant material was authenticated and stored at the Herbarium of the Botany Department of the Federal University of Juiz de Fora, MG, Brazil. The leaves (20 g) were dried at room temperature, powdered in a knife mill and firstly defatted, by maceration, using n-hexane. Based on previous studies that shown *A. chica* is rich in flavonoids and deoxyanthocyanidins [50], CHCl_3_ was chosen as solvent for extraction. Then, the powered material was extracted, by maceration, using CHCl_3_ as solvent. Following extraction, the solvent was filtered into a round bottom flask and removed under vacuum, at 40 °C, using a rotary evaporator to yield 2 g of the crude chloroform extract of *A. chica*, which was used in all assays.

### Antioxidant activity

The free-radical scavenging activity was measured by the [2,2’-azinobis-(3-ethylbenzothiazoline-6-sulfonate)] (ABTS+) assay according with da Cruz et al. (2020) [54] with some modifications. The ABTS+ radical cation stock solution was prepared by mixing 7 mM ABTS+ with potassium persulfate (140mM), incubated at room temperature in the dark for 16 h. The ABTS+ was then diluted in ethanol until reached OD 0.700 ± 0.020 at 734nm. CEAC (125 μg/mL; 250 μg/mL; 500 μg/mL and 1000 μg/mL) was mixed with ABTS+ in 96-well plates. Absorbance was read at 415 nm using a microplate reader (Robonik®). Trolox was used as reference at concentrations ranging from 0.1 to 2 mM and the results were expressed as Trolox equivalents.

### Cell culture

Four established breast cell lines were used throughout this study: (i) MCF 10A, non-tumorigenic, grown in DMEM/F-12 medium supplemented with 10 μg/mL of EGF, 0,25 μg/mL of Hydrocortisone and 10 μg/mL of insulin; (ii) MCF-7, ER-positive BC, maintained in RPMI-1640 medium; (iii) T-47D, ER-positive BC, also kept in RPMI-1640 medium supplemented with 10 μg/mL of insulin, and (iv) MDA-MB-231, triple-negative BC, cultured in L15 medium. The cell lines were obtained from American Type Culture Collection and confirmed to be free of mycoplasma contamination.

Tamoxifen-resistant MCF7 cells (MCF-7/TamR) were obtained after prolonged and continuous exposure of the MCF7 lineage to Tam (ranging from 0.1 μM to 1.0 μM) for a period of three months, as previously described [51–54].

All strains were supplemented with 10% of FBS, and 50 μg/mL of gentamycin, and kept in culture at 37° C in an atmosphere of 5% CO2 (Thermo Scientific™ Forma Series 3 Water Jacketed CO_2_ Incubator). For the cell line MDA-MB-231 the flasks were kept closed, free from CO_2_.The medium was changed on alternate days, until cell reached 80-90% confluence, when they were used in subsequent experiments.

### Cell viability assay

The cell viability was evaluated by MTT reduction following previously instructions published by [55], with minor modifications. The four cell lines were cultured and, after confluence and trypsinization, 1x10^4^ cells of each lineage were seeded in 96-well microplates with proper culture conditions for 24 h. Subsequently, cells were treated with 7 μg/mL, 15 μg/mL, 30 μg/mL, 62 μg/mL, 125 μg/mL, 250 μg/mL, 500 μg/mL and 1000 μg/mL of CEAC for 24 and 48 h. MTT solution (5mg/mL) was then added, incubated for 4 h, and the supernatant was carefully discarded. The insoluble formazan crystals produced by intracellular dehydrogenase was solubilized with DMSO, and the absorbance of each well was determined at 570 nm using Automatic Elisa Plate Reader (IndiaMART, DD Bioinfotech / Nathupura, New Delhi).

Each sample was measured in triplicate, and each experiment was repeated three times (*n*= 3). The mean OD of the treated cells was compared to the mean OD of the control wells (treated with vehicle only-DMSO) [55]. Wells with complete medium, without cells, were considered as blank. Cell viability was reported according to Formula (F1) = [(Absample-Absblank)/(AbsDMSO-Absblank)*100].

The half maximal inhibitory concentration (IC_50_) was calculated by non-linear regression from a dose-response curve between the compound concentration and percent growth inhibition [56], using the GraphPad Prism 6.0 software (GraphPad Software, San Diego, CA, USA). Selective indexes (SI) were also calculated using the Formula (F2) = SI = IC_50_-MCF 10A/IC_50_-BC cells. Values of SI ≥ 2.0 are considered significant [57].

The CEAC was selective to ER-positive BC cells. For this reason, the viability of T-47D and MCF-7 cells was further evaluated, as described above, after treatment, for 48 h, with CEAC at 1000 μg/mL; 1 μM and 2 μM of Tam [58, 59], alone. Cells were also treated with CEAC associated with Tam at 1 μM and 2 μM simultaneously or with one compound followed by the other. Finally, the viability of MCF-7/TamR cells was evaluated under the same conditions mentioned above.

### qPCR analysis

Total RNA was isolated from MCF-7, T-47D and MCF-7/TamR cells treated with CEAC (1000 μg/ mL), Tam (1μM), and CEAC associated with Tam for 48 h, using Trizol® reagent (Invitrogen). The protocol was followed according to the supplier’ instructions. Cells treated with DMSO were included as control. q-PCR was carried out to evaluate the capacity of CEAC and Tam to modulate the transcriptional levels of *ESR1*, *ESR2* and *AR* in ER-positive BC cell lines. The quality of extracted RNA was verified by electrophoresis on 1.5% agarose gel, stained with GelRed 1x (Uniscience), as well as by the reason of the spectrophotometric readings at 260 and 280 nm (Nanodrop 1000-ThermoFischer). First-strand cDNA was synthetized as previously described [60].

q-PCR was carried out on StepOnePlus Systems (Applied Biosystems), using 5.0 μM of specific primers designed for each gene as follows: *ESR1* - F: CTAACTTGCTCTTGGACAGGAAC / R: GATTTGAGGCACACAAACTCCTC; *ESR2* - F: GGGAATGGTGAAGTGTGGCT / R: TCATGTGTACCAACTCCTTGTCGG; *AR:* F: CATGTGGAAGCTGCAAGGTCT / R: GTGTAAGTTGCGGAAGCCAGG [38, 60]. Transcripts were quantified by ΔCq method after relative standard curve optimization with 5.0 μL of Power SYBR Green PCR Master Mix (Applied Biosystems, Carlsbad, CA, USA) and 2.0 μL cDNA. All data were normalized by β-2-microglobulin (*β2M*) gene (F: CCTGCCGTGTGAACCATGT / R: GCGGCATCTTCAAACCTCC) [60].

### Epithelial Tumor Test (ETT)

Four concentrations of CEAC were defined (2.5, 5.0, 10.0 and 20.0 μg/μL) to be used in *in vivo* assay, and ETT was performed according to the methods proposed by Costa and contributors [61]. Heterozygotic larvae *wts* +/+ *mwh* were obtained from the cross between virgin females *wts*/*TM*_*3*_, *Sb*^*1*^ [45] with males *mwh*/*mwh* [61]. Third-instar larvae (72 ± 4 h) were submitted to a chronic treatment for about 48h. The *D. melanogaster* strains were kept under optimal laboratory conditions (25 ± 4 °C and 65% RH) in BOD-type chamber (Model: SL224, SOLAB – Equipamentos para Laboratórios, São Paulo, SP, Brazil).

At first, the toxicity test (TX) was carried out to assess the lethal concentration of CEAC for *D. melanogaster*. Larvae of third-instar (72 ± 4 h) were counted and placed in separate tubes containing 1.5 g of culture medium (mashed potatoes) [44, 62] for *D. melanogaster* with 5.0 mL of different concentrations of CEAC, alone (2.5, 5.0, 10.0 e 20.0 μg/μL) or in association with DOX (CEAC at 2.5, 5.0, 10.0 μg/μL, and DOX at 0.4 mM). The number of surviving flies per treatment were counted and provided an indicator of the toxicity of the compound [48].

Based on TX test, we performed ETT in post-treatment format [43, 48, 61]. The larvae were pre-treated with DOX at 0.4 mM to induce tumors and, after 6 h, the third stage larvae (72 ± 4 h) were washed and subjected to chronic treatment with CEAC at 2.5, 5.0, 10.0 μg/μL. Three controls were included: (i) negative control reverse osmosis water; (ii) solvent control with tween 80 1% (v/v) used to dilute CEAC; and (iii) positive control with DOX at 0.4mM. All experiments were conducted in quadruplicate.

Emerging adult flies from the different treatments were collected and kept in ethanol 70%. Only adult flies without the chromosome balancer *TM*_*3*_, *Sb*^*1*^ were analyzed, which can be differentiated phenotypically by the absence of truncated bristles [43, 45 48]. Tumors can be detected in all segments of the fly and *D. melanogaster* stands out for being an experimental model useful in genetic toxicology tests, as well as in studies of DNA repair processes.

### Statistical analyses

Data were expressed as the mean ± standard deviation (SD) from three independent experiments. For the MTT assay, differences between the viability across cells lines were determined using one-way analysis of variance (ANOVA) and the Tukey HSD post hoc. Gene expression data were compared through Student’s independent t-test. Statistical comparisons of survival rates (TX) of *D. melanogaster* were performed using the Chi-squared (X^2^) test for ratios of independent samples. All the above results were analyzed using the GraphPad Prism 7.0 (GraphPad Software Inc., La Jolla, CA, EUA). Statistical significance was considered when *p* < 0.05.

The *A. chica* carcinogenic and/or anticarcinogenic potential, evaluated in ETT test, was determined by the Mann, Whitney and Wilcoxon nonparametric *U* test, with α=0.05 level of significance, using Prophet 5.0 (Phophet Software).

## Results

### Antioxidant potential and cytotoxicity

**Fig. 1:**
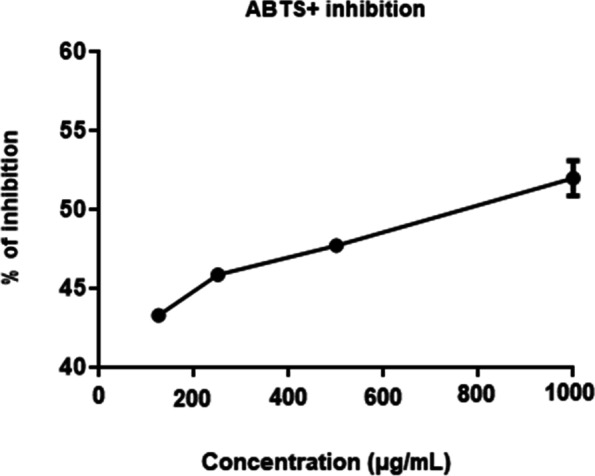
Antioxidant activity of the chloroform extract of *Arrabidaea chica* (CEAC) evaluated by the ABTS^+^ method. The tested concentrations of CEAC were 125 μg/mL, 250 μg/mL, 500 μg/mL and 1000 μg/mL. The results are represented relative to Trolox, as reference standard

The antioxidant potential of CEAC was determined by the radical scavenging activity using the ABTS^+^ method and the results expressed as % of inhibition relative to Trolox as reference standard. The ABTS^+^ test showed radical scavenging activities in a dose dependent manner, and, in the highest concentration of CEAC (1000 μg/mL), the ABTS^+^ radicals were inhibited by 51.92%, showing the antioxidant potential of the extract (Fig. 1).

**Fig. 2:**
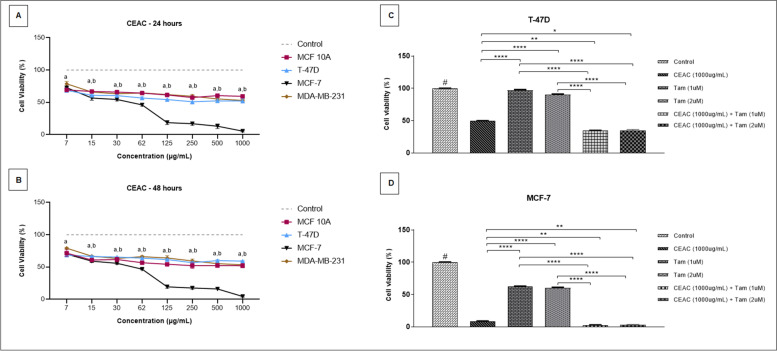
Cytotoxic effects of chloroform extract of *Arrabidaea chica* (CEAC) on human luminal breast cancer cells T-47D, MCF-7. The non-tumorigenic cell line MCF 10A and the triple-negative breast cancer cell MDA-MB231 were included in this study. Cells treated with DMSO (diluent) were used as control. Treatments were performed on the four cell lines with different concentrations of CEAC for 24 (**A**) and 48 hours (**B**). Cell viability rates of luminal breast cancer cell lines were also recorded after treatment with CEAC and Tamoxifen (Tam) for 48 hours in T-47D (**C**) and MCF-7 (**D**) cell lines. Cells were treated with CEAC and Tam in isolation and combined. Data are presented as mean ± S. D of three independent experiments. Significance was calculated by one-way ANOVA, and Tukey’s post hoc test. # treatments with DMSO differed from all treatments with CEAC, *p* < 0.0001. a: treatment in MCF-7 differed from treatment in MCF 10A. b: treatment in MCF-7 differed from treatment in T-47D. (*p < 0.05, **p < 0.01, ***p < 0.001, ****p < 0.0001)

Subsequently, we evaluated the cytotoxic effect of CEAC on human breast cell lines including tumorigenic (T-47D, MCF-7, and MDA-MB-231) and non-tumorigenic (MCF 10A) lineages through MTT assay, performed for 24 (Fig. 2A) and 48 hours (Fig. 2B). The cellular behavior was similar in both treatments in which the viability of the MCF 10A lineage was maintained at around 57% for all tested concentrations. The cytotoxicity of CEAC was significantly higher for MCF-7 cells when compared to control (cells treated with DMSO, only) and to other cell lines, mainly in concentrations above 62 μg/mL. The IC_50_ of CEAC on MCF-7 cells was estimated at 8 μg/mL, with SI of 180.87.

As the viability of the non-tumorigenic cell MCF 10A did not differ across treatments and CEAC was selective for luminal breast tumor cells, the concentration of 1000 μg /mL was used for further experiments in treatments for 48 hours in T-47D and MCF-7 cell lines, which express ER. Tam is widely used as ER antagonist, and it is the first line therapy for ER-positive BC. However, 40% of women receiving Tam develop resistance, which comprises treatment and patient’s survival [63]. Therefore, we compared the cellular effects of CEAC with Tam (1 μM and 2 μM) in isolated and combined treatments. All treatments differed from the control with diluent and the behavior of both cells was not different according to the Tam concentration. The effects were similar for 1 μM and 2 μM of Tam.

For the T-47D cell line (Fig. 2C), treatment with CEAC alone significantly reduced viability when compared to treatments with Tam alone. However, the cytotoxic effect was even greater when CEAC was combined with Tam. The viability of T-47D cells after treatment with CEAC was 53% and with CEAC + Tam (1 μM) was 34%. Tam also significantly decreases the viability of the MCF-7 cells (Fig. 2D). However, corroborating with our data, the cytotoxicity of CEAC for the MCF-7 was even more expressive and significantly higher when the extract was combined with Tam. Therefore, the viability of MCF-7 cells after treatment with CEAC was 7% and with CEAC+Tam (1 μM) was 4%The lowest concentration of Tam was then defined for further molecular assays.

### Modulation of hormone receptors transcriptional levels

**Fig. 3:**
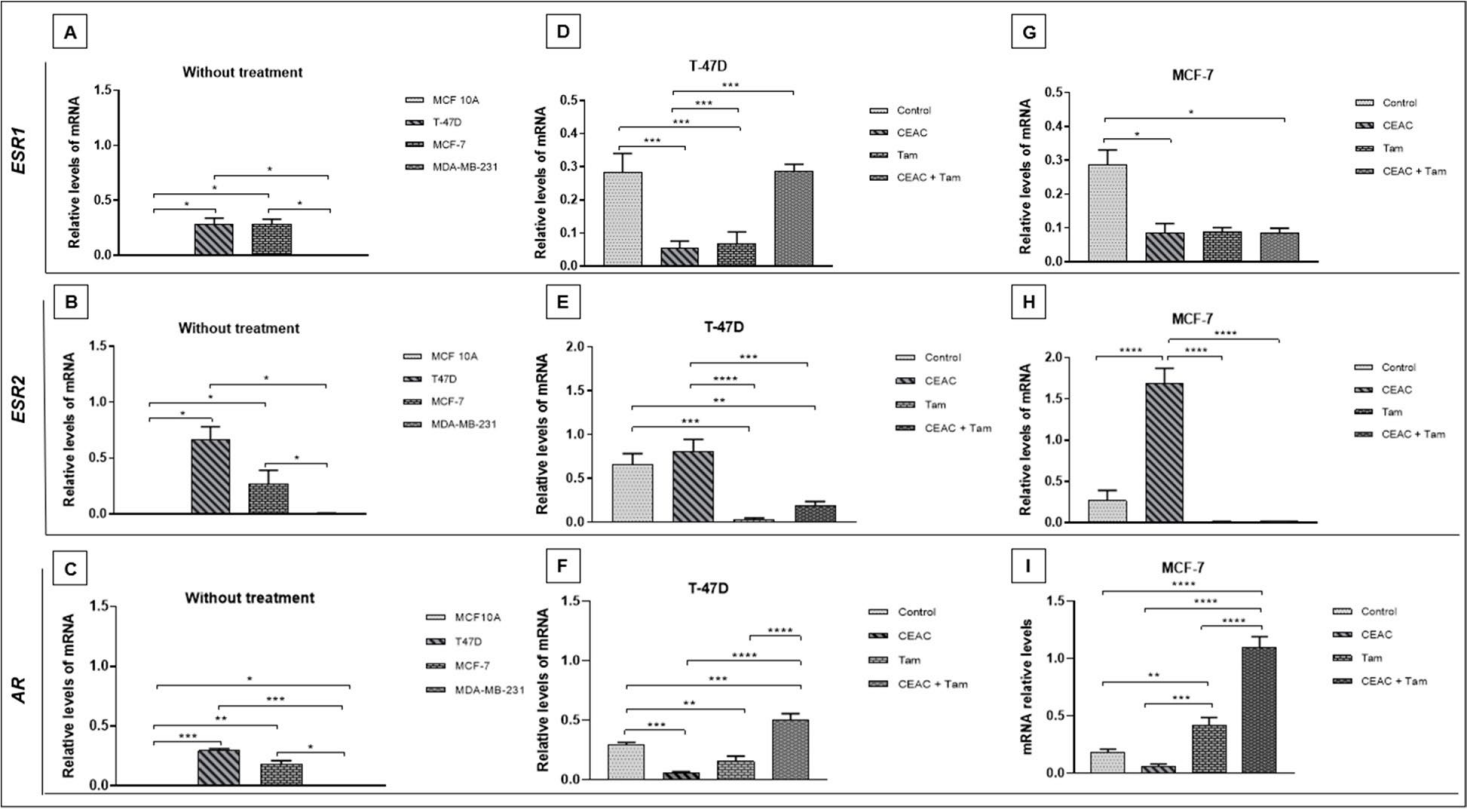
Relative levels of hormone receptors transcripts after treatment with the chloroform extract of *Arrabidaea chica* (CEAC) and Tamoxifen (Tam). Gene expression levels were recorded without treatment in MCF 10A, T-47D, MCF-7 and MDA-MB231 cell lines (**A**, **B**, **C**). T-47D (**D**, **E**, **F**) and MCF-7 (**G**, **H**, **I**) cell lines were treated with 1000 μg /mL of CEAC, Tam (1 μM) and CEAC + Tam (1000 μg /mL, and 1 μM, respectively) for 48 h. The relative expression levels of the genes encoding for Estrogen Receptor alpha (*ESR1*), Estrogen Receptor beta (*ESR2*), and Androgen receptor (*AR*) were quantified by the comparative Cq method. * *p* <0,05, ** *p* <0,01, *** *p* <0,001 e **** *p* <0,0001

The expression of the *ESR1*, *ESR2* and *AR* genes was quantified in T-47D and MCF-7 cell lines treated with CEAC (1000 μg /mL) and Tam (1μM), alone and with CEAC + Tam (combined treatment) for 48 hours (Fig. 3). As expected, MCF 10A and MDA-MB231 do not express hormone receptors (Fig. 3A-C).

In the T-47D cells (Fig. 3D) the expression of *ESR1* decreased by 5.6-fold (*p* <0.01) and 4.2-fold (*p* <0.001) after treatments with CEAC or Tam, respectively, when compared to control (cells treated with diluent). When CEAC was associated with Tam, the expression of *ESR1* increased, and differed only from the treatment with the extract alone. Regarding the *ESR2* gene (Fig. 3E), although slightly higher, its expression did not differ from the control in T-47D cells treated with CEAC. *ESR1* levels decreased significantly when T-47D cells were treated with Tam alone (22.7-fold) and with CEAC + Tam (3.5-fold), compared to control. When treatment with CEAC was compared with Tam, *ESR2* transcripts were 27.8-fold higher after treatment with CEAC. For both receptors, the combined treatments did not promote a significant difference in gene expression compared to treatment with Tam alone. Analysis of *AR* expression (Fig. 3F) revealed that the transcripts were differentially modulated between experiments, with higher *AR* transcriptional levels in the combined treatment of CEAC + Tam. Interestingly, CEAC + Tam treatment led to an increase in AR expression and a rescue of *ESR1* expression that, accordingly to Park and collaborators, would represent a BC with better outcome. Moreover, when Tam was associated to CEAC, there was a partial rescue of *ESR2* expression that, accordingly to the above researchers, could avoid the establishment of Tam resistance [37].

For the MCF-7 cells CEAC was responsible for promoting a decrease in *ESR1* expression (alone or combined with Tam) (Fig. 3G), and an increase in *ESR2* transcripts, which was 6.2-fold higher when compared to control (*p* <0.01), 105.6-fold higher when compared to treatment with Tam (*p* <0.01), and 99.4-fold higher when compared to CEAC + Tam treatment (*p* <0.001) (Fig. 3H). Finally, the *AR* transcripts were downregulated after treatment of MCF-7 with CEAC, upregulated after treatment with Tam alone and upregulated at higher levels with CEAC + Tam treatment (Fig. 3I).

**Fig. 4:**
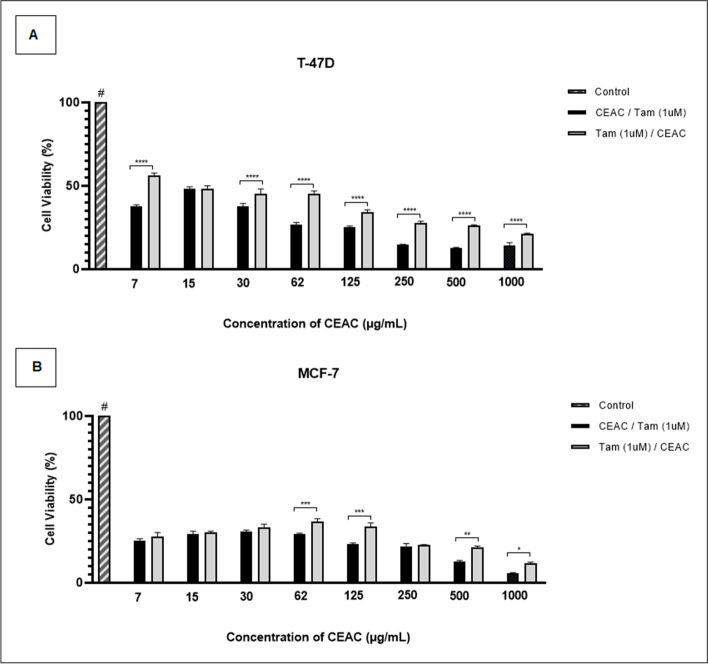
Cell viability rates of luminal breast cancer cell lines after alternate treatment with chloroform extract of *Arrabidaea chica* (CEAC) and Tamoxifen (Tam) for 48 hours. T-47D (**A**) and MCF-7 (**B**) cell lines were treated with CEAC followed by Tam (1μM) or with Tam (1μM) followed by CEAC (1000 μg/mL). Data are expressed as means ± SD, *n* = 3. Significance (*p < 0.05, **p < 0.01, ***p < 0.001, ****p < 0.0001) was calculated by one-way ANOVA, and Tukey’s post hoc test. # treatments with DMSO (control) differed from all treatments with CEAC, *p* < 0.0001

As CEAC modulated the expression of *ESR2* we tested the effect of the extract before or after treatment with Tam at 48 hours intervals (Fig. 4). Interestingly, the viability of the T-47D (Fig. 4A) and MCF-7 (Fig. 4B) cell lines was significantly compromised. Furthermore, the initial treatment with CEAC (1000 μg/mL) followed by Tam (1 μM) showed a greater reduction in viability. For T-47D cells the viability reached 14% and for the MCF-7 lineage 3%.

### Cytotoxicity and modulation of hormone receptors in MCF-7/TamR

**Fig. 5:**
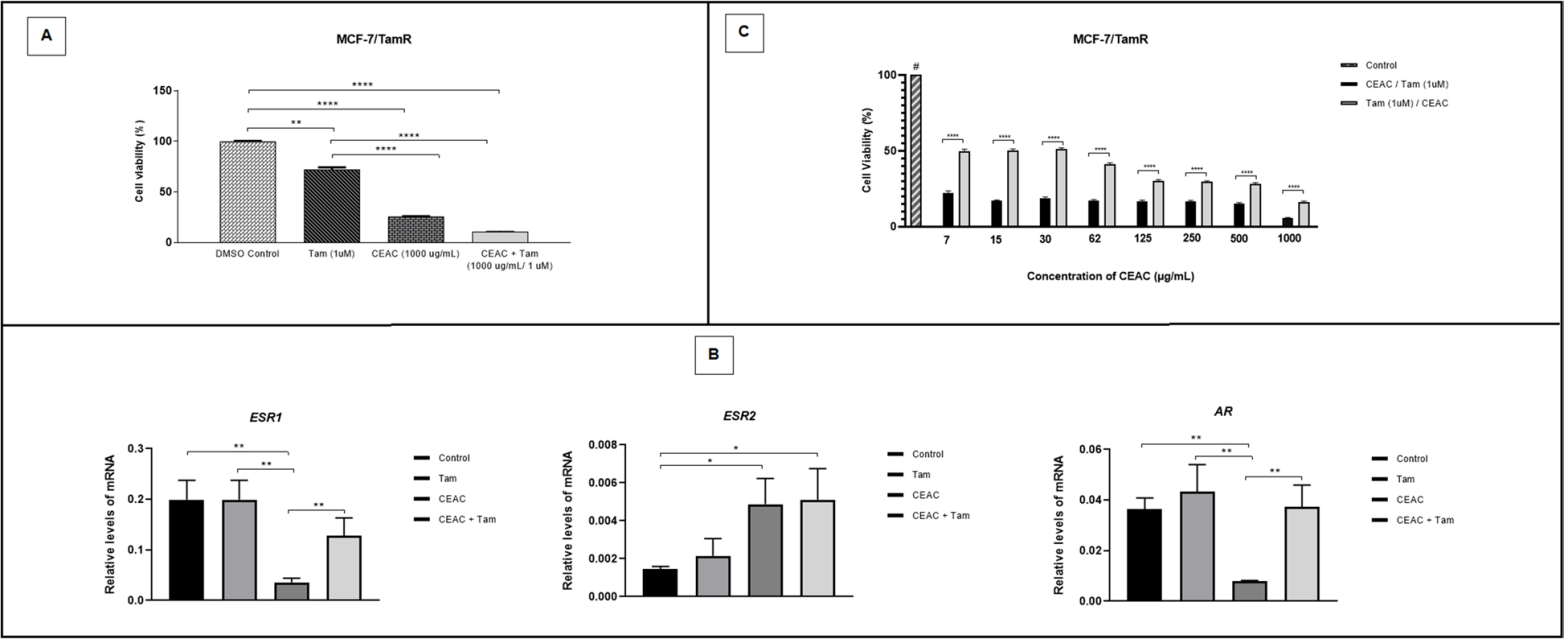
Effect of chloroform extract of *Arrabidaea chica* (CEAC) and Tamoxifen (Tam) on tamoxifen-resistant MCF7 cells (MCF-7/TamR). Cell viability of MCF-7/TamR cells after treatment with CEAC (1000 μg/mL), Tam (1μM) or with CEAC + Tamfor 48 hours (**A**). Relative levels of hormone receptors transcripts after treatment with CEAC, Tam or CEAC + Tam. Gene expression was recorded through Cq method (**B**). MCF-7/TamR cell lines were also treated with CEAC followed by Tam (1μM) or with Tam (1μM) followed by CEAC (1000 μg/mL) and the viability evaluated by MTT (**C**). Data are expressed as means ± SD, *n* = 3. Significance (*p < 0.05, **p < 0.01, ***p < 0.001, ****p < 0.0001) was calculated by one-way ANOVA, and Tukey’s post hoc test. # treatments with DMSO differed from all treatments, *p* < 0.0001. *ESR1:* Estrogen Receptor alpha, *ESR2:* Estrogen Receptor beta (*ESR2*), and *AR:* Androgen receptor

Treatment with CEAC alone reduced the viability of the MCF-7/TamR lineage by 90% (Fig. 5A), differing significantly from control and treatment with Tam. However, the profile of resistant cells treated with CEAC + Tam did not differ from the cytotoxic effect observed for the extract alone. As for hormone receptors (Fig. 5B), CEAC was responsible for decreasing the expression of *ESR1* and *AR* in these cells and for increasing *ESR2* gene transcripts, compared to the control. For CEAC + Tam treatments, only *ESR2* transcripts were upregulated and Tam treatments did not differ from control.

We also investigated the effect of the extract before or after treatment with Tam at 48-hour intervals (Fig. 5C). The viability of the MCF-7/TamR lineage was significantly decreased under all conditions. However, previous treatment with CEAC (1000 μg/ml) followed by Tam (1 μM) promoted a significantly greater reduction in the viability of resistant cells.

### Effect of CEAC on *D. melanogaster*

*D. melanogaster* has been used for more than 50 years as a model for human diseases related to alterations in replication, DNA repair, translation, drug metabolism and in toxicological research. Among the several advantages of using *D. melanogaster* stands out its genetic and metabolic similarity with humans and its ability to enzymatically activate promutagens and procarcinogens *in vivo* [48].

**Fig. 6:**
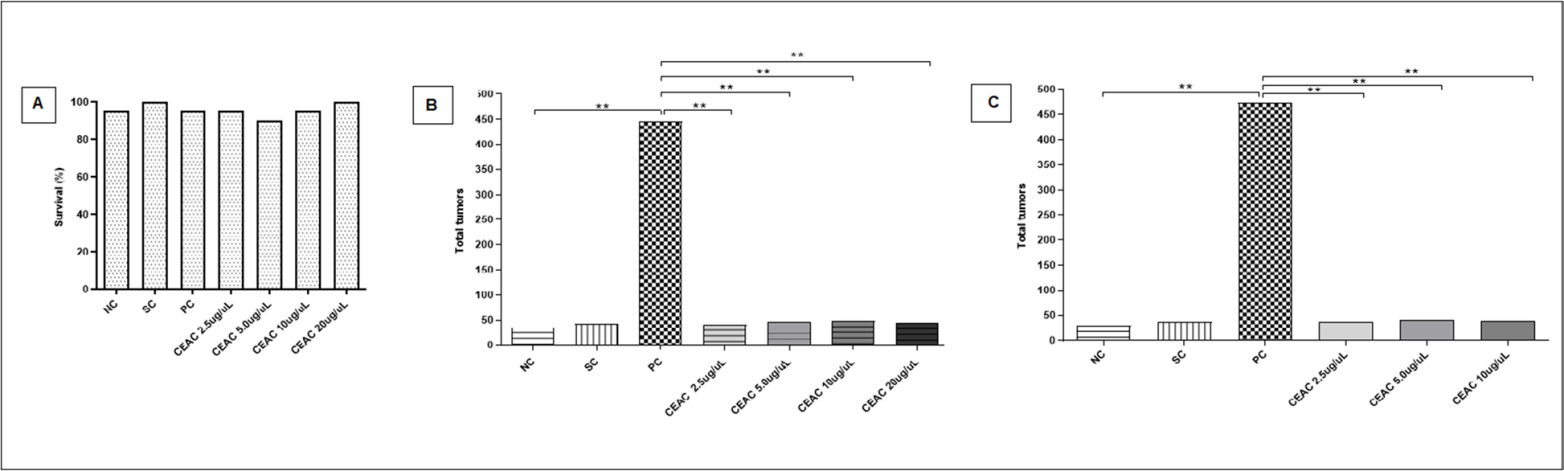
*In vivo* assays performed with *D. melanogaster* treated with the chloroform extract of *Arrabidaea chica* (CEAC). Percentage of survival of *D. melanogaster* treated with CEAC (**A**). Treatments were conducted with CEAC (2.5, 5.0, 10.0 and 20.0 μg/μL). Flies, heterozygous for the *Warts* tumor suppressor gene, were further treated with different concentrations of CEAC (2.5, 5.0 and 10.0 μg/μL) and total tumors were recorded to demonstrate the carcinogenic (**B**) effect of CEAC. To demonstrate the anticarcinogenic effect of CEAC (**C**) flies were pre-treated with Doxorubicin (DOX 0.4 mM) and, subsequently, chronic treated for 48h with different concentrations of the extract. The frequency of tumors was analyzed in different segments, and significance was calculated by the Mann-Whitney Test. **Values considered significant when compared to the positive control (DOX 0.4 mM). *Values considered different from the negative control (*P* < 0.05). NC, negative control (osmosis reverse water). SC, solvent control (Tween 80 1%). PC, positive control (DOX 0.4mM). NC: Negative control (reverse osmosis water); SC: Solvent control (Tween 80 at 1%); PC: Positive control (DOX 0.4 mM)

At first, CEAC toxicity was investigated to define the concentrations to be used in ETT assay. The percentage of surviving flies from treatment with tween 80 at 1% (negative control) and with DOX 0.4 mM (positive control) did not differ statistically from treatment with reverse osmosis water (negative control). Regarding treatment of CEAC (2.5, 5.0, 10 and 20 μg /μL) (Fig. 6A), the extract was not significantly toxic to *D. melanogaster* larvae when compared to the negative control and therefore was used in subsequent experiments.

CEAC at concentrations of 2.5, 5.0 and 10.0 μg/μL was then assessed by ETT (Fig. 6B-Table 1), and no statistically significant carcinogenic effect of the extract was identified when compared to the negative control. As expected, the solvent control (Tween 80 at 1%) did not differ statistically from the negative control (reverse osmosis water); and DOX 0.4 mM (positive control) generated significantly more tumors than the negative control. This concentration of DOX was based on previous studies that demonstrated the induction of epithelial tumors in *D. melanogaster* treated with DOX 0.4 mM [38, 43].

**Table 1:** Tumor clone frequency observed in *D. melanogaster*, heterozygous for the *Warts* tumor suppressor gene. Flies were treated with osmosis reverse water (negative control); Doxorubicin 0.4mM (DOX, positive control), and different concentrations of chloroform extract of *Arrabidaea chica* alone (CEAC 2.5, 5.0 and 10.0 μg/μL) to observe the carcinogenic effect of the extract. To assess the anticarcinogenic effect of CEAC, flies were pre-treated with Doxorubicin (DOX 0.4 mM) and, subsequently, chronic treated for 48h with different concentrations of the extract

Treatment			Number of Flies	Number of tumors							Frequency	
	CEAC (μg/μL)	DOX (mM)		Eye	Head	Wing	Body	Leg	Halter	Total		
Carcinogenic effect of CEAC	0	0	200	0.00 (00)	0.00 (00)	0.02 (04)	0.15 (30)	0.01 (02)	0.00 (00)	36	(0.18)	
	0	0.4	200	0.24 (49)	0.05 (10)	0.17 (35)	1.51 (302)	0.24 (48)	0.01 (02)	446	(2.23) ^a^	
	2.5	0	200	0.01 (02)	0.00 (00)	0.03 (07)	0.14 (28)	0.02 (04)	0.00 (00)	41	(0.20) ^ns^	
	5.0	0	200	0.02 (04)	0.03 (06)	0.02 (04)	0.11 (23)	0.04 (09)	0.00 (00)	46	(0.23) ^ns^	
	10	0	200	0.00 (01)	0.03 (06)	0.02 (05)	0.13 (27)	0.04 (09)	0.00 (00)	48	(0.24) ^ns^	
**Treatment**			**Number of Flies**	**Number of tumors**							**Frequency**	**Inhibition (%)**
	**CEAC (μg/μL)**	**DOX (mM)**		**Eye**	**Head**	**Wing**	**Body**	**Leg**	**Halter**	**Total**		
Anticarcinogenic effect of CEAC	0	0	200	0.00 (00)	0.00 (01)	0.02 (05)	0.09 (19)	0.02 (04)	0.00 (00)	29	(0.14)	
	0	0.4	200	0.25 (51)	0.05 (10)	0.21 (42)	1.54 (309)	0.29 (59)	0.01 (03)	474	(2.37) ^a^	
	2,5	0.4	200	0.01 (02)	0.01 (03)	0.03 (06)	0.09 (18)	0.04 (08)	0.00 (01)	38	(0.19) ^b^	92%
	5,0	0.4	200	0.02 (04)	0.01 (02)	0.04 (08)	0.10 (20)	0.03 (07)	0.00 (00)	41	(0.20) ^b^	91.4%
	10	0.4	200	0.01 (02)	0.03 (06)	0.01 (02)	0.10 (20)	0.04 (09)	0.00 (00)	39	(0.19) ^b^	91.8%

Finally, the anticarcinogenic potential of CEAC is represented in Fig. 6C. The individuals were pre-treated with DOX (0.4 mM) for a period of 6 hours and later treated with different concentrations of CEAC (2.5, 5.0 and 10.0 μg/μL). The findings for all three concentrations evaluated differed significantly from the positive control, with a significant reduction in the frequency of total number of tumors in CEAC-treated flies. The inhibition percentages of each concentration (2.5, 5.0 and 10.0 μg/μL) were 92%, 91.4% e 91.8%, respectively (Table 1).

The frequency of tumors was analyzed in different segments, and significance was calculated by the Mann-Whitney Test. ^a^Values considered different from the negative control (*p* <0.05). ^ns^ Values considered not significant when compared to the negative control. ^b^Values considered significant when compared to the positive control (DOX 0.4 mM). Frequency: number of tumors / fly

## Discussion

The present study reveals CEAC as an estrogenic and androgenic modulator, potentially promising in the treatment of luminal BC and tamoxifen-resistant cells. Luminal breast tumors express ER and are responsive to endocrine therapy. Estrogen mediates breast cell growth [38, 55] and is associated with etiology of BC being an important target for ER antagonists, such as Tam [59]. However, despite the good prognosis, treatment for ER-positive breast tumors remains challenging, as therapeutic resistance has compromised patients’ survival [57, 58]. Hormonal status has been shown to be decisive in clinical outcome and understanding how different compounds alter the expression of receptors has been crucial for BC management.

Natural products are versatile and perform important antitumor activities, as they act in different pathways, blocking tumorigenesis and controlling the progression of transformed cells [32]. *A. chica* protective effects involve the reduction of ROS levels and lipid peroxidation supporting the antioxidant potential of CEAC identified in the present study using the ABTS^+^ method. In addition, this species increases of collagen content during the healing process, with analgesic properties through the inhibition of cyclooxygenases [33, 64–67] also demonstrated that the plant extract alone, or associated with vincristine, decreased serum transaminases levels, oxidative stress, and hematological toxicity. Our work is pioneer in demonstrating the role *of A. chica* in the modulation of hormone receptors.

In *in vitro* assays with breast cell lines, CEAC substantially inhibited the viability of MCF-7 cells, with SI of 180.87. As MCF-7 cells are ER-positive, the results raised the question about the extract's ability to modulate hormone receptors. The ER response is the result of a balance between the signaling pathways of two divergent isoforms ER-α and ER-β [68]. ER-α expression is associated with the development, growth and metastasis of BC [69], and ER-β has been described as tumor suppressor. In addition, ER-β expression independently predicts better disease-free survival in patients treated with Tam [70]. In the present study, *ESR1* transcriptional levels were downregulated after treatment of MCF-7 cells with CEAC, and *ESR2* gene expression was upregulated. This behavior was also observed in treatments performed in tamoxifen-resistant cells. Therefore, the observed cytotoxicity can be related to the extract's ability to modulate ERs, which led us to the hypothesis about the possibility of the compound being combined with Tam in the treatment of luminal BC cell lines.

When CEAC was combined with Tam, the treatment was slightly more cytotoxic to the T-47D and MCF-7 strains. However, for ERs, there was no difference in the expression of these genes compared to treatments with Tam alone. For *AR*, also evaluated in the present study, the modulatory effects were more evident, and similar between the T-47D and MCF-7 with lower mRNA levels when cells were treated with CEAC and higher levels when treated with CEAC + Tam. AR stimulates cell proliferation, promotes metastasis and favors resistance to endocrine therapy [71–73]. Previous studies indicated that one third of all patients develop resistance to Tam, even though ER-α positivity remains unchanged [74, 75]. In addition, *in vitro* experiments confirmed a low expression of ER-β in tumors resistant to Tam [53]. Our results demonstrated decreased expression of *ESR2* when cells were treated with Tam alone. In the treatment with CEAC + Tam, this profile was maintained in MCF-7 and a partial rescue of *ESR2* was obtained in T47-D cells. This partial rescue could indicate a possible effect of CEAC in diminishing the development of Tam resistance in T47D cells. Moreover, Tam + CEAC treatment leads to the maintenance of *ERS1* and *AR* levels which, accordingly to Park and collaborators, would represent a BC with better outcome [37]. Interestingly, the effect of Tam on *AR* expression was different in MCF-7 cells compared to T47D cells. In T47 D cells, Tam decreased *AR* expression while in MCF7 cells there was an increase in *AR* expression. Since T47D are considered a more Tam resistant cell line compared to MCF-7 cells [76], this effect on *AR* expression could be responsible for the different Tam sensitivity. On the other hand, treatment with CEAC alone achieves one of the main objectives of endocrine therapy by increasing the expression of *ESR2* and decreasing that of *ESR1* and *AR* in MCF-7 cells and in MCF-7/TamR.

In this context, enhanced ER-β signaling can modulate ER-α and AR signaling without complete ablation of hormones [77–82]. We suggest that the different effects between isolated and combined treatments may result from competition and / or antagonisms between CEAC and Tam, which needs to be further investigated. We therefore suggest the treatment with CEAC as complementary to Tam, once, the expression and ER-β can sensitize cells to Tam [83–86]. This effect was proven for T-47D cells and MCF-7/TamR. The initial treatment with CEAC and then with Tam substantially reduced the viability of T-47D cells to 14% and to 11% in MCF-7/TamR. This behavior may be associated with CEAC's ability to modulate *ESR2* [87–89]*.* However, further studies are needed to define the therapeutic design and to evaluate the inclusion of CEAC in the treatment of women resistant to Tam. Finally, our results demonstrated a different response profile between the two cell lines of luminal BC. Therefore, we suggest a deeper study of the molecular characterization of luminal breast tumors, as these subtypes may require different therapeutic regimens, which may be limiting for the prognosis of patients.

Regarding the *in vivo* assays, CEAC (2.5, 5.0, 10.0 and 20.0 μg/μL) did not show significant toxicity for *D. melanogaster,* validating the safety of these concentrations. In the ETT assay, CEAC did not demonstrate carcinogenic effect at the three concentrations tested (2.5, 5.0 and 10.0 μg/μL). Previous studies have reported that aqueous, butanolic and chloroform extracts of *A. chica* did not cause mutagenic effect in strains of *Salmonella*. Even with metabolic activation, the chloroform extract did not show mutagenic effect suggesting the absence of phytochemicals capable of inducing frameshift mutation [43, 62, 90, 91]. Although mutagenicity is an inherent factor in carcinogenicity, both mutagens and non-mutagens compounds can generate transformed cells inducing different cancer hallmarks, including cellular proliferation [43, 62, 92]. Thus, the absence of a mutagenic effect corroborates and reinforces our results on the absence of CEAC carcinogenic potential, supporting the safety of the compound.

Table 1 shows the post treatment model, in which the tumor was induced with DOX (0.4mM) so that the larvae were subjected to chronic treatment with different concentrations of CEAC (2.5, 5.0 and 10.0 μg/μL). The *wts* gene is maintained in heterozygosity in stock of *D. melanogaster* in the presence of a chromosomal balancer (*TM3*). The loss of heterozygosity in the cells of the larval imaginal disc leads to the proliferation of cell clones as tumors in adults [43, 93]. DOX at 0.4 mM has a carcinogenic effect on somatic cells of *D. melanogaster* [44, 48, 94], which was reversed with CEAC treatment, showing its ability to reduce epithelial tumors. In this context, we suggest that CEAC increases the antioxidant potential of *D. melanogaster* cells, helping to reduce the damage caused by metabolites generated by DOX, which mimics the genomic damage that cause tumors. The synchronism between DNA replication, with the repair of damage, and the progression of the cell cycle ensure the integrity of the genome avoiding mutations and rearrangements in the DNA [95, 96]. Chromosomal mutations or aberrations affect oncogenes and tumor suppressor genes leading to the malignant transformation [48, 97]. Thus, genomic instability is associated with serious pathological disorders such as cancer [44, 48, 98–101]. In addition, CEAC induces apoptosis of damaged cells during the embryonic development of the larvae, with consequent reduction of tumors in adults. Therefore, we also suggest that, in the post-treatment, the anticarcinogenic effect of CEAC is due to the increased activity of proteins involved in repair pathways related to the tumor suppressor gene *Warts*, which promoted the control of cell proliferation and reduction of the formation of epithelial tumors. Importantly, we use the ETT test that assesses the toxicity, mutagenicity and carcinogenicity of different compounds [98, 102–105], based on phenotypic effects. However, it has been shown that aqueous and ethanolic extracts of *A. chica* inhibit inflammatory and angiogenic processes [89, 106, 107] and that its ethanolic extract also reduces the lipid oxidative stress marker malondialdehyde [67, 108]. In the present work we also suggest its role in the modulation of hormone receptors.

## Conclusion

Our study confirmed the antioxidant activity of CEAC, emphasizing its important role in carcinogenesis. The bioassays on breast tumor cells demonstrated the antiproliferative activity of the extract, being selective to the BC luminal cells. CEAC modulated the expression of *ESR1*, *ESR2* and *AR*. In *in vivo* tests, CEAC was not toxic to *D. melanogaster*, demonstrated anticarcinogenic action, and controlled tumor formation in DOX-treated flies.

As far as we are aware, this is the first study of the hormone modulatory effect of CEAC on BC cells and the anticarcinogenicity effect of CEAC. It will be important to confirm our results in other animal models, as well as further experiments that seek to discover additional mechanisms of this extract in BC.

## Data Availability

The datasets generated and/or analyzed during the current study are available from the corresponding author on reasonable request.

## Abbreviations

AR: Androgen receptor
BC: Breast cancer
CEAC: Chloroform extract *Arrabidaea chica*
DOX: Doxorubicin
ER: Estrogen receptor
ESR1: Estrogen receptor alpha
ESR2: Estrogen receptor beta
ETT: Epithelial tumor test
Her2: Human epidermal receptor
NR: Nuclear receptor
PgR: Progesterone receptor
ROS: Reactive oxygen species
SERM: Selective estrogen receptor modulators
Tam: Tamoxifen
TX: Toxicity test
wts: Tumor suppressor gene
